# Case Report of Optic Disc Drusen with Simultaneous Peripapillary Subretinal Hemorrhage and Central Retinal Vein Occlusion

**DOI:** 10.1155/2014/156178

**Published:** 2014-12-02

**Authors:** David Zhiwei Law, Francine Pei Lin Yang, Stephen Charn Beng Teoh

**Affiliations:** ^1^National Healthcare Group Eye Institute, Tan Tock Seng Hospital, 11 Jalan Tan Tock Seng, Singapore 308433; ^2^Vision Performance Centre, Military Medicine Institute, Singapore Armed Forces, Block 27 Medical Drive, DSO Building Lower Kent Ridge Road, Singapore 117510; ^3^Eagle Eye Centre Novena, 38 Irrawaddy Road, Mount Elizabeth Novena Specialist Centre No. 08-22/23/24, Singapore 329563

## Abstract

A 52-year-old Chinese gentleman presented with right eye floaters and photopsia over one week. His visual acuities were 20/20 bilaterally. Posterior segment examination showed a right eye swollen optic disc and central retinal vein occlusion (CRVO) associated with an area of subretinal hemorrhage adjacent to the optic disc. Fundus fluorescein (FA) and indocyanine green angiographies (ICGA) of the right eye did not demonstrate choroidal neovascularization (CNV), polypoidal choroidal vasculopathy (PCV), or retinal ischemia. Ultrasound B-scan revealed optic disc drusen (ODD). In view of good vision and absence of CNV, he was managed conservatively with spontaneous resolution after two months. Commonly, ODD may directly compress and mechanically rupture subretinal vessels at the optic disc, resulting in peripapillary subretinal hemorrhage, as was likely the case in our patient. Mechanical impairment of peripapillary circulation also results in retinal ischemia and may trigger the development of choroidal neovascularization (CNV) and/or polypoidal choroidal vasculopathy (PCV), leading to subretinal haemorrhage. Compromise in central venous outflow with increased retinal central venous pressure from the direct mechanical effects of enlarging ODD results in central retinal vein occlusion (CRVO). Patients with subretinal hemorrhage and CRVO from ODD should be monitored closely for the development of potentially sight-threatening complications.

## 1. Introduction

Optic disc drusen (ODD) may be associated with subretinal hemorrhage as a result of direct mechanical compression and rupture of subretinal vessels at the optic disc, resulting in peripapillary subretinal hemorrhage [1–5]. Mechanical impairment of peripapillary circulation also results in retinal ischemia and release of vascular endothelial growth factor (VEGF), which may trigger the development of choroidal neovascularization (CNV) and/or polypoidal choroidal vasculopathy (PCV), leading to subretinal haemorrhage [3, 6–10]. Compromise in central venous outflow with increased retinal central venous pressures from direct mechanical effects of enlarging ODD results in central retinal vein occlusion (CRVO) [1, 2, 7, 10, 11]. We present a case of peripapillary subretinal hemorrhage with simultaneous CRVO arising from the direct mechanical compression of ODD on peripapillary and retinal vessels, in the absence of CNV and PCV.

## 2. Case Presentation

A 52-year-old Chinese gentleman with no significant vascular risk factors presented with right eye floaters for a 1-week duration associated with photopsia without blurring of vision or metamorphopsia. He had low myopia (−2.0DS OU) and no history of trauma to his eyes.

**Figure 1:**
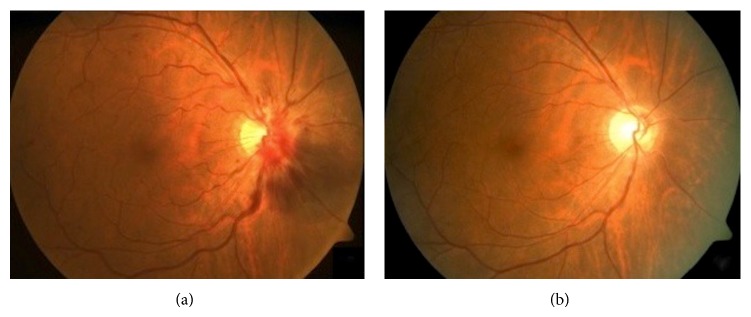
(a) Intraretinal hemorrhages over a right swollen optic disc associated with peripapillary hemorrhages, an area of subretinal hemorrhage inferonasally and venous tortuosity consistent with CRVO. (b) Spontaneous resolution of right optic disc swelling, peripapillary subretinal hemorrhage, and CRVO after two months.

His best-corrected visual acuities (BCVA) were 20/20 OU with intraocular pressures of 15 mmHg bilaterally on Goldmann applanation tonometry (GAT) and no relative afferent pupillary defect (RAPD). Anterior segment examinations were unremarkable. Posterior segment examination showed right optic disc swelling which was more pronounced nasally, flame-shaped optic disc hemorrhages, venous tortuosity, and extensive flame hemorrhages without vasculitis, consistent with CRVO. Although the Disc-Macula/Disc-Disc (DM/DD) ratio on fundal photography was measured to be 2.44 (normal) and nonindicative of optic disc anomalies for example, small optic discs associated with optic nerve hypoplasia (ONH), there was marked filling of the physiological cup. In addition, there was an area of subretinal hemorrhage of 1.5 disc-diameter inferonasal to the optic disc (Figure 1(a)). Fundal examination of the left eye was normal.

## 3. Results

**Figure 2:**
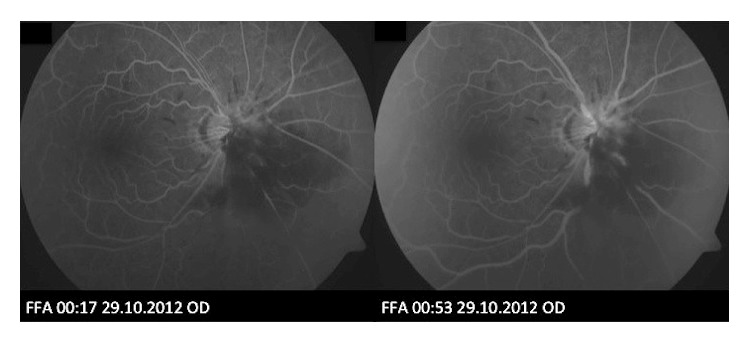
Fluorescein angiography (FA) of the right eye that does not demonstrate peripapillary leakage to suggest choroidal neovascularization (CNV) or hemorrhage. There is also no capillary fall-out to suggest retinal ischemia.

**Figure 3:**
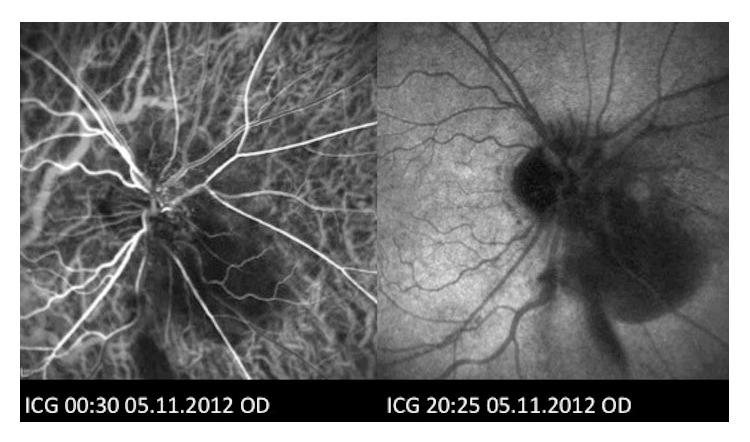
Indocyanine green angiography (ICGA) of the right eye showing absence of hyperfluorescent polyps to suggest the presence of polypoidal choroidal vasculopathy (PCV).

**Figure 4:**
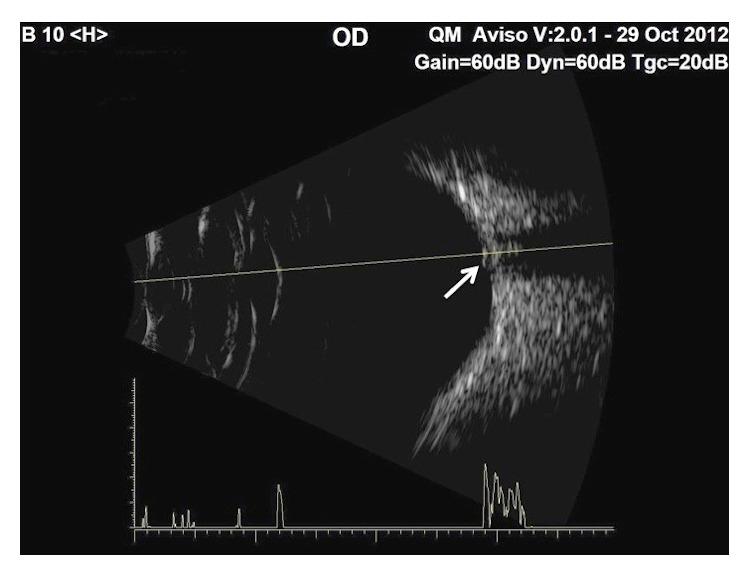
B-scan (low gain) of the right optic disc showing moderately high reflectivity consistent with surface ODD (arrow).

In view of CRVO, investigations for vascular risk factors were performed which showed a blood pressure of 130/80 mmHg, fasting blood glucose level of 5.4 mmol/L, and hypercholesterolaemia. Fluorescein angiography (FA) demonstrated blocked fluorescence over the area of the subretinal hemorrhage but no capillary fallout or leakage (Figure 2). Indocyanine green angiography (ICGA) did not reveal any hyperfluorescent hot spots suggestive of PCV (Figure 3). B-scan ultrasound revealed moderately high reflectivity over the elevated optic disc consistent with surface ODD (Figure 4). A diagnosis of right ODD with simultaneous complications of peripapillary subretinal hemorrhage and CRVO from direct mechanical compression of peripapillary and retinal vessels was made. In the absence of CNV, PCV, and macular oedema as well as preservation of good BCVA, he was managed conservatively with close observation. Follow-up at 2 months after presentation showed spontaneous resolution of his right optic disc swelling, subretinal hemorrhages, and CRVO (Figure 1(b)).

## 4. Discussion

ODD are calcified hyaline-like deposits in the optic nerve head resulting from calcific degeneration and deposition of calcium, amino acids, ribonucleic acids, and iron anterior to the lamina cribrosa [7, 11, 12]. They arise from alteration in axoplasmic flow of ganglion cells and often occur in eyes with small optic discs (horizontal diameter <1.68 mm, vertical diameter <1.94, and area <2.6 mm^2^), increased DM/DD ratio (>2.94) [13, 14], and small scleral canals [2, 7, 12, 15–18]. They occur in 0.4–20.4% of the population and may be bilateral (67–91%) and familial (autosomal dominant) [6, 7, 10–12, 15, 17, 19–21]. They show a predominance for females (58–71%) and Caucasians [3, 7, 12, 20]. ODD are often benign and asymptomatic. Commonly found in the nasal aspect of the optic disc, they may present with pseudopapilledema, especially if buried beneath the optic disc surface [11].

Uncommonly, they may manifest with more significant visual disturbances occurring as a result of disturbances to the retinal nerve fiber layer (RNFL) [3]. Reported complications include visual field defects (concentric constriction, enlargement of the blind spot, and arcuate scotomas) [1, 7, 10–12, 15–17, 20, 21], hemorrhages (2–10%) in 3 layers, within the RNFL localized over the optic disc, deep within the peripapillary subretinal layer extending from the disc to the surrounding retina and over the optic disc within extension into the vitreous [1, 3, 4, 7, 10, 20, 22, 23], CNV, serous maculopathy, and nonarteritic ischemic optic neuropathy (NAAION) [6, 16]. In 11% of cases, vascular anomalies such as arterial or venous loops, optociliary shunt vessels, cilioretinal arteries, retinal-choroidal collaterals, pronounced venous tortuosity, and dilatation, as well as abnormal early branching, for example, bi-/trifurcation of vessels, are present [3, 4, 6, 7, 10, 12, 16, 20].

ODD often mimic papilledema and must be distinguished from true papilledema arising from more sinister causes [1–4, 6, 7, 10, 20, 24]. As such, a thorough clinical examination and use of adjunctive investigation tools such as ultrasound B-scan, FA, IGA, and Computed Tomography (CT) scan may be necessary. In contrast to papilledema, ODD is characterized by an absence of disc hyperemia, venous congestion, and exudates with the drusen, as well as the presence of spontaneous venous pulsation and filling of the physiological cup. Typically, there is also elevation of the optic disc which is confined to the nasal aspect and does not extend beyond the disc margins [11].

Superficial peripapillary vessels of small optic discs and scleral canals associated with ODD are vulnerable to direct mechanical distortion. Enlarging ODD, which are concretions with sharp edges, may further rupture these vessels, resulting in superficial RNFL hemorrhages over the optic disc and peripapillary areas [1–5]. Direct mechanical compression and rupture of veins arising from within the optic disc may also lead to peripapillary subretinal hemorrhage beneath the retinal pigment epithelium (RPE). This can spread towards the macula, potentially affecting central vision [3]. The central retinal vein traverses the lamina cribrosa, and progressive enlargement of ODD causing increased mechanical compression of this vessel further disrupts retinal venous outflow and elevates central retinal venous pressure, resulting in central retinal vein occlusion (CRVO) [1, 2, 7, 10, 11]. Superficial and subretinal hemorrhages arising from the direct mechanical compressive effects of ODD are typically self-limiting, as was the case in our patient. In the absence of CNV and PCV, no treatment is required for ODD associated with subretinal hemorrhage and CRVO. Regardless, subretinal hemorrhage may progress to affect central vision and patients must be closely observed for the development of complications such as CNV and NAAION [3, 6, 16].

Less commonly, ODD may also cause retinal ischemia and release of vascular endothelial growth factor (VEGF) which stimulates CNV formation and subretinal hemorrhage [3, 6–10]. In the absence of subfoveal progression, submacular hemorrhage, or serous macular detachment, active treatment of ODD-associated CNV with modalities such as focal laser photocoagulation, photodynamic therapy, or anti-VEGF therapy is often not indicated [3, 20, 25].

## 5. Conclusion

The subretinal hemorrhage in our patient is likely due to the direct mechanical compressive effects of the ODD on the walls of superficial and subretinal peripapillary vessels. Progressive enlargement of the ODD further erodes these peripapillary vessels, causing subretinal hemorrhage. Compression of the central retinal vein by ODD at the lamina cribrosa leads to disruption to venous outflow and increased retinal central venous pressure, which accounts for the findings of concomitant CRVO. Although treatment of the subretinal hemorrhage is unnecessary in the absence of CNV and PCV, regular reviews are required to monitor for the development of complications such as progression of subretinal hemorrhage to the macula as well as development of CNV and NAAION that may be potentially sight-threatening and are amenable to treatment [3, 6–10, 16, 24]. Importantly, ODD presenting with pseudopapilledema must be distinguished from true papilledema.

## References

[1] Sanders T. E., Gay A. J., Newman M. (1970). Drüsen of the optic disk-hemorrhagic complications. *Transactions of the American Ophthalmological Society*.

[2] Rubinstein K., Ali M. (1982). Retinal complications of optic disc drusen. *British Journal of Ophthalmology*.

[3] Romero J., Sowka J., Shechtman D. (2008). Hemorrhagic complications of optic disc drusen and available treatment options. *Optometry*.

[4] Dhingra N., Prasad S. (2003). Optic disc drusen. *Journal of Postgraduate Medicine*.

[5] Jane W. C., Jane W. C. (2007). Chapter 8: congenital disc anomalies. *Optic Nerve Disorders—Diagnosis and Management*.

[6] Aumiller M. S. (2007). Optic disc drusen: complications and management. *Optometry*.

[7] Auw-Haedrich C., Staubach F., Witschel H. (2002). Optic disk drusen. *Survey of Ophthalmology*.

[8] Diduszyn J. M., Quillen D. A., Cantore W. A., Gardner T. W. (2002). Optic disk drusen, peripapillary choroidal neovascularization, and POEMS syndrome. *American Journal of Ophthalmology*.

[9] Harris M. J., Fine S. L., Owens S. L. (1981). Hemorrhagic complications of optic nerve drusen. *American Journal of Ophthalmology*.

[10] Davis P. L., Jay W. M. (2003). Optic nerve head drusen. *Seminars in Ophthalmology*.

[11] Brodrick J. D. (1973). Drusen of the disc and retinal haemorrhages. *British Journal of Ophthalmology*.

[12] Calvo-Gonzalez C., Santos-Bueso E., Diasz-Valle D. (2006). Optic nerve drusen and deep visual fields defects. *Archives of the Spanish Society of Ophthalmology*.

[13] Wakakura M., Alvarez E. (1987). A simple clinical method of assessing patients with optic nerve hypoplasia. *Acta Ophthalmologica*.

[14] Zeki S. M., Dudgeon J., Dutton G. N. (1991). Reappraisal of the ratio of disc to macula/disc diameter in optic nerve hypoplasia. *British Journal of Ophthalmology*.

[15] Younan N. M., Francis I. C. (2003). Progressive visual failure in an eye with optic disc drusen and an orbital mass. *Journal of Neuro-Ophthalmology*.

[16] Sarkies N. J. C., Sanders M. D. (1987). Optic disc drusen and episodic visual loss. *British Journal of Ophthalmology*.

[17] Turgut B., Kaya M. K., Demir T. (2010). An atypical case of optic disk drusen with nerve fiber layer thickening. *Eye Brain*.

[18] Gili P., Flores-Rodríguez P., Yangüela J., Orduña-Azcona J., Martín-Ríos M. D. (2013). Evaluation of optic disc size in patients with optic nerve head drusen using fundus photography. *Journal of Optometry*.

[19] Katz B. J., Pomeranz H. D. (2006). Visual field defects and retinal nerve fiber layer defects in eyes with buried optic nerve drusen. *American Journal of Ophthalmology*.

[20] Morris R. W., Ellerbrock J. M., Hamp A. M., Joy J. T., Roels P., Davis C. N. (2009). Advanced visual field loss secondary to optic nerve head drusen: case report and literature review. *Optometry*.

[21] Beck R. W., Corbett J. J., Thompson H. S., Sergott R. C. (1985). Decreased visual acuity from optic disc drusen. *Archives of Ophthalmology*.

[22] Henkind P., Alterman M., Wie G. N., Cant J. S. (1972). Drusen of the optic disc and sub-pigment epithelial haemorrhage. *The Optic Nerve*.

[23] Sanders T. E., Gay A. J., Newman M. (1971). Hemorrhagic complications of drusen of the optic disk. *American Journal of Ophthalmology*.

[24] Şahin A., Cingü A. K., Ari Ş., Çinar Y., Çaça I. (2012). Bilateral optic disc drusen mimicking papilledema. *Journal of Clinical Neurology*.

[25] Delyfer M. N., Rougier M. B., Fourmaux E., Cousin P., Korobelnik J.-F. (2004). Laser photocoagulation for choroidal neovascular membrane associated with optic disc drusen. *Acta Ophthalmologica Scandinavica*.

